# Hombres que trabajan a través de aplicaciones móviles en Brasil: reflexiones desde la salud ocupacional

**DOI:** 10.18294/sc.2024.4588

**Published:** 2024-03-21

**Authors:** João Vitor Antunes Lins dos Santos, Thais Janaína Wenczenovicz, Adriana Remião Luzardo, Jeferson Santos Araujo

**Affiliations:** 1 Enfermero, especialista en Oncología y Hematología. Estudiante de Maestría, Programa de Posgrado Interdisciplinar em Ciencias Humanas, Universidade Federal da Fronteira Sul, Erechim, Brasil. lins.joaovitor2@gmail.com Universidade Federal da Fronteira Sul Programa de Posgrado Interdisciplinar em Ciencias Humanas Universidade Federal da Fronteira Sul Erechim Brazil lins.joaovitor2@gmail.com; 2 Posdoctora en Educación. Profesora, Programa de Posgrado Interdisciplinar em Ciencias Humanas, Universidade Federal da Fronteira Sul, Erechim, Brasil. t.wencze@terra.com.br Universidade Federal da Fronteira Sul Programa de Posgrado Interdisciplinar em Ciencias Humanas Universidade Federal da Fronteira Sul Erechim Brazil t.wencze@terra.com.br; 3 Doctora en Enfermería. Profesora, Programa de Posgrado en Enfermería, Universidade Federal da Fronteira Sul, Chapecó, Brasil. adriana.luzardo@uffs.edu.br; 4 Doctor en Enfermería. Profesor, investigador, Programa de Posgrado Interdisciplinar en Ciencias Humanas, Programa de Posgrado en Enfermería, Universidade Federal da Fronteira Sul, Chapecó, Brasil. jeferson.araujo@uffs.edu.br Universidade Federal da Fronteira Sul Programa de Posgrado Interdisciplinar en Ciencias Humanas Programa de Posgrado en Enfermería Universidade Federal da Fronteira Sul Chapecó Brazil jeferson.araujo@uffs.edu.br

**Keywords:** Masculinidad, Salud Ocupacional, Riesgos Laborales, Salud del Hombre, Mercado de Trabajo, Brasil, Masculinity, Occupational Health, Occupational Risks, Men’s Health, Job Market, Brazil

## Abstract

El trabajo a través de aplicaciones móviles surgió como una alternativa para muchos hombres brasileños durante la crisis económica y el aumento del desempleo. Sin embargo, estos trabajadores operan de manera informal, sin relación laboral, lo que genera precariedad laboral y falta de derechos laborales. Desde un abordaje teórico-reflexivo, este artículo analiza la intersección entre la salud ocupacional, la plataformización del trabajo y los desafíos específicos que enfrentan los hombres repartidores de aplicaciones, especialmente durante la pandemia de covid-19. Las categorías reflexivas se dividieron en dos secciones temáticas: “La inserción laboral a través de aplicaciones móviles en Brasil y el modelo de trabajo mediante algoritmos” y “hombres, salud y motocicletas: la salud ocupacional y el comportamiento masculino en el trabajo a través de aplicaciones de entrega”. Se examina la expansión de este tipo de trabajo en el contexto socioeconómico brasileño, la falta de seguridad y protección laboral para estos trabajadores, así como los riesgos enfrentados, especialmente durante la pandemia, en el marco de una cultura masculina.

## Abstract (en)

Work through mobile applications emerged as an alternative for many Brazilian men during the economic crisis and rising unemployment. However, these workers operate informally, without a formal employment relationship, leading to labor precariousness and a lack of workers’ rights. Using a theoretical-reflexive, this study analyzes the intersection between occupational health, the platformization of work, and the specific challenges faced by male delivery app workers, especially during the COVID-19 pandemic. Reflective categories are divided into two thematic sections: “Labor insertion through mobile applications in Brazil and the algorithmic work model” and “men, health, and motorcycles: occupational health and male behavior in app-based delivery work.” The study examines the expansion of this type of work in the Brazilian socio-economic context, the lack of security and labor protection for these workers, as well as the risks faced, particularly during the pandemic, within a male-centric culture.

## INTRODUCCIÓN

En 2020, el mundo fue impactado por el covid-19, una variante del coronavirus humano que rápidamente alcanzó proporciones globales. En marzo del mismo año, la Organización Mundial de la Salud (OMS) declaró el estado de pandemia, una emergencia de salud pública y de interés internacional^1^. Los efectos de esta pandemia afectaron severamente varios sectores de la economía y, en países en desarrollo como Brasil, una amplia gama de trabajadores informales buscó otras formas de obtener ingresos. Así, en el escenario brasileño de recesión económica y aumento del desempleo, una parte significativa de la población excluida del mercado formal encontró otras formas de subsistencia que, en ocasiones, los alejan de los derechos laborales y sociales^2^.

Los avances de las tecnologías de la información y comunicación han causado grandes cambios en el mundo y en las relaciones laborales. Un fenómeno global que surge en este contexto es la llamada “plataformización del trabajo”^3^, que representa una nueva fase del trabajo precarizado. Este modelo reorganiza las relaciones laborales con la idea central de dispersar el trabajo sin perder el control sobre él, transformando a los trabajadores en autogestores subordinados. Aunque no estén contratados formalmente, muchos individuos actúan como colaboradores de aplicaciones de entrega o movilidad urbana para llevar a cabo sus funciones laborales y garantizar su subsistencia^4^.

Con la emergencia de este nuevo sistema, los trabajadores pasaron a ser reclutados, gestionados y controlados a través de aplicaciones, y la lógica del trabajo formal se ha vuelto cada vez más escasa. En este contexto, la narrativa de ser su propio jefe despertó premisas de un sistema de trabajo flexible, creando un nuevo enfoque en la relación entre el individuo y el entorno laboral. Esta relación coexiste con la exigencia de un perfil de trabajador proactivo, capaz de satisfacer las demandas esperadas, pero que, al mismo tiempo, tiene la autonomía de ser gestor de su propio horario laboral^5^.

En el contexto brasileño, Manzano y Krein^6^ destacan un creciente número de trabajadores vinculados a plataformas digitales que, en 2021, totalizaban 1,4 millones de usuarios activos. Estas empresas operan principalmente en la movilidad urbana y en la plataforma de entregas, consolidando una nueva forma de gestionar, organizar y controlar el trabajo, lo que se ha convertido en una tendencia global después del proceso de desindustrialización y el fortalecimiento de los sectores de servicios.

Se observa que en estas profesiones hay una fuerte presencia de trabajadores de sexo masculino, siendo que aproximadamente cinco de cada seis conductores de aplicaciones son hombres^6^. Al examinar este número, se percibe una compleja relación entre dos mundos: el motociclismo y las ideas de masculinidad^7^^,^^8^. Esta relación se configura tanto objetiva como subjetivamente, influenciada por estándares masculinos que forman el estereotipo negativo de imprudencia, audacia, irresponsabilidad, incivilidad y placer por emociones fuertes^8^ que presentan una notable coincidencia con valores y comportamientos históricamente asociados al universo masculino.

El predominio de hombres en este tipo de trabajo puede estar directamente asociado a la construcción de la identidad masculina, que a menudo se relaciona con ideales tradicionales de masculinidad, como el dominio en las relaciones de género, el control financiero y el papel de proveedor del hogar^9^. De esta manera, es importante destacar que la concepción de masculinidad puede ejercer una influencia directa en el comportamiento de los hombres en el tránsito.

Esta construcción del estereotipo masculino influye profundamente en la forma en que utilizan y perciben sus cuerpos dentro de la sociedad en la que viven. Adoptan un modelo de masculinidad idealizado por el entorno social en el que están insertos, compartiendo creencias y comportamientos que refuerzan las características consideradas masculinas^10^^,^^11^. Esto puede llevar a decisiones y actitudes que ponen en riesgo la salud, ya que a menudo se descuida la valoración de características consideradas femeninas, como el autocuidado y la atención a la vida en el tráfico^8^.

Sin embargo, estar expuestos a estos riesgos puede perjudicar la garantía de derechos laborales y de protección social. Al unirse a la plataforma de manera espontánea, estos individuos vinculados como repartidores y conductores carecen de seguridad social en el mercado laboral, ya que no hay un vínculo formal entre ambas partes. Estas transformaciones instauran condiciones que dependen cada vez menos de actividades intelectuales, llevando a una desvalorización y depreciación de la acción humana^2^. Así, los trabajadores quedan completamente desprovistos de derechos en lo que respecta a su actividad laboral, además de que su remuneración se basa en una lógica algorítmica^3^^,^^4^.

En cuanto a la salud ocupacional, los cambios ocurridos en los últimos dos años de pandemia han generado una reconfiguración y un rediseño del mapa de accidentes y enfermedades en el trabajo. En esta misma ola de nuevos perfiles epidemiológicos y sociales, el aumento exponencial de trabajadores que operan a través de aplicaciones también se convierte en un foco de atención para esta área de la salud. Uchôa de Oliveira^12^ destaca que si la plataformización del trabajo “representa una nueva forma de organización del trabajo, la salud del trabajador se verá afectada directamente por este proceso”.

En este escenario, la salud de estos hombres queda a merced de la transferencia de responsabilidades. Al mismo tiempo que trabajan, asumen los riesgos y costos y, en consecuencia, se alejan de cualquier derecho y protección laboral. Así, las nuevas modalidades de actividades laborales fragmentan la cadena productiva y permiten la sobreexplotación de trabajadores que realizan jornadas más extensas y enfrentan mayores inseguridades y vulnerabilidades^13^. La plataformización del trabajo actúa como una forma de organización del trabajo al mismo tiempo que aleja a los trabajadores de la garantía de derechos y se convierte en un foco para otras esferas político-sociales como, en este caso, la salud del trabajador.

### Plataformización del trabajo

El concepto de “plataformización del trabajo” surge de la creciente influencia de las plataformas digitales en la organización y ejecución de las actividades laborales. Según Abílio^4^, la intersección entre los avances tecnológicos y la economía de plataformas impacta directamente en la dinámica del trabajo, introduciendo nuevas formas de empleo, relaciones laborales y demandas para las personas trabajadoras. El contrato de trabajo, que formaliza el vínculo con la empresa, asume ahora la configuración de un contrato de adhesión. La naturaleza de la relación de subordinación se vuelve más informal, y este proceso de informalización implica la ausencia de predeterminaciones claras o estables en cuanto a la jornada laboral, remuneración o derechos.

Para la autora, el fenómeno de la plataformización^3^ abarca diversas perspectivas y puede ser examinado a través de diferentes enfoques, como la datificación, la vigilancia, la financiarización y la función de los algoritmos, entre otros. El elemento que atraviesa todas estas aproximaciones es la administración algorítmica del trabajo, caracterizada por la vigilancia continua e imperceptible sobre el trabajador, la recopilación de datos y su integración al capital, la disponibilidad constante del trabajador sin obligaciones recíprocas y la concepción del trabajo como un simple factor de producción, desvinculado de responsabilidades asociadas a su reproducción y garantías laborales.

Este modo de trabajo crea una dualidad entre la dispersión en la producción y la centralización del control laboral. Como resultado, existen varios trabajadores distribuidos geográficamente por Brasil, todos sujetos a las políticas de una misma entidad empresarial que ejerce el comando de forma centralizada. Este manejo algorítmico transforma a la empresa en algo más significativo que una simple intermediaria, revelando una dinámica de subordinación y control sobre el trabajo^3^^,^^4^. Y bajo esta dinámica, la empresa no solo define el valor del servicio prestado por el trabajador al consumidor y su remuneración, sino que también mantiene un control absoluto sobre la distribución del trabajo y las reglas que lo rigen^14^.

Esta naturaleza multifacética de la plataformización del trabajo explora no solo las implicaciones económicas, sino también los efectos sociales y psicológicos de esta transformación. En la plataformización del trabajo, la flexibilidad y la informalidad características de este nuevo paradigma laboral afectan las condiciones de trabajo, los derechos de los trabajadores y las prácticas relacionadas con la salud ocupacional^3^^,^^4^.

Como destacan Abílio *et al*.^3^, los trabajadores tienden a generar sus propias metas salariales y someterse a jornadas de trabajo extenuantes, lo que resulta en perjuicios para su salud y bajo rendimiento laboral debido a la fatiga. Otra problemática es que requiere la regulación de las emociones durante las constantes interacciones interpersonales en el ambiente profesional. Las consecuencias de esta demanda incluyen agotamiento emocional e insatisfacción laboral, impactando especialmente a los proveedores de servicios, cuyas responsabilidades demandan sostener emociones positivas, a pesar de situaciones estresantes como el tráfico urbano en las grandes metrópolis^14^. En este sentido, al adoptar un enfoque algorítmico, se acentúa la manipulación del trabajador a través de las herramientas de evaluación del conductor por parte del cliente. Estas herramientas son utilizadas para reforzar el control de la aplicación sobre el desempeño del conductor, con el monitoreo de la calidad del servicio prestado y el estímulo a la productividad laboral^4^. Estos elementos se utilizan para integrar la tecnología al entorno laboral, ampliar la eficiencia y mejorar el ritmo de producción de los trabajadores^14^.

Sin embargo, al intensificarse la subutilización de habilidades y de calificaciones, sumado a la remuneración inadecuada y las jornadas de trabajo por debajo de lo deseado -condiciones que son comunes a las plataformas de trabajo- se deteriora el compromiso organizacional, generando malestar psicológico e insatisfacción laboral para los proveedores de servicios, cuyas habilidades a menudo superan las exigidas por el trabajo realizado^14^. En este escenario, las plataformas retienen la mayor parte de las ganancias generadas por el servicio prestado por los trabajadores, creando una brecha entre el trabajador, su reconocimiento y sus derechos laborales ya que, al registrarse en la plataforma, el trabajador es considerado solo un socio de la empresa y no un empleado registrado formalmente^3^^,^^4^.

### Masculinidades

Según Raewyn Connel^9^, las masculinidades son intrínsecas a la cultura del ser y las reconoce como una configuración práctica que abarca la posición de los hombres en las relaciones de género. En este contexto, existe una masculinidad culturalmente hegemónica, un tipo ideal que sirve como punto de referencia. Para alcanzar este estatus, los hombres deben ejercer la cultura del patriarcado guiados por afirmaciones como “*el hombre no se enferma*”, “*el hombre sostiene a su familia y necesita tener una remuneración mayor que la mujer*”. Sin embargo, no todos los hombres adoptan este papel, pudiendo expresar diferentes manifestaciones de lo masculino en el mismo contexto social y en situaciones específicas, como en el sustento financiero, al adoptar masculinidades de complicidad, resistencia, subordinación y marginación^9^.

Para la autora, la masculinidad es la forma en que los individuos asumen su posición de poder dentro de la sociedad y los efectos de estas prácticas en su experiencia corporal y cultural. Se trata de una construcción sociohistórica dentro de la singularidad del hombre en su entorno de formación y que ahora reproduce en su vida cotidiana. Así, las discusiones sobre el significado de ser hombre y, consecuentemente, sobre la identidad masculina, no solo dan forma a los comportamientos interpersonalmente esperados socialmente, sino que también ejercen influencia en la percepción de los cambios en la vida de los hombres y en la forma en que se relacionan socialmente^9^.

Para fortalecer estas identidades, los hombres son instigados socialmente a perpetuar jerarquías y privilegios que emanan del patriarcado. Esta hegemonía está asociada a las prácticas relacionales y a los significados discursivos que se relacionan con las desigualdades de género entre hombres y mujeres, entre masculinidad y feminidad, y entre distintas manifestaciones de masculinidad^15^.

En la dinámica de las prácticas relacionales de género, las manifestaciones de masculinidad son influenciadas por la interacción de varios elementos que componen las identidades individuales, tales como indicadores sociales de clase, raza, etnia, generación y sexualidad. Esta interacción resulta en representaciones dinámicas y diversificadas, que Separavich y Oliveira^16^ describen como procesos sociales complejos. Entre estos, destacamos los vinculados al trabajo y la subsistencia, que desempeñan un papel crucial en la manera en que los hombres expresan y experimentan la masculinidad, alejándose de la idealización socialmente valorada. Este distanciamiento es resultado de la capacidad de los hombres para reinterpretar los marcadores sociales que fundamentan la construcción de la masculinidad dominante^17^. 

Considerando que las regulaciones laborales no garantizan formas de trabajo seguras para estos colaboradores informales que constantemente se encuentran en situaciones de riesgo en el desempeño de sus actividades laborales, este artículo tiene como objetivo reflexionar sobre la intersección entre salud ocupacional, plataformización del trabajo y los desafíos específicos enfrentados por los hombres motociclistas de aplicaciones, especialmente durante la pandemia de covid-19.

## METODOLOGÍA

Este estudio adoptó un enfoque teórico-reflexivo para analizar críticamente la bibliografía reciente que aborda el aumento del trabajo informal, con un enfoque especial en los hombres repartidores en motocicleta. Este fenómeno impacta significativamente los derechos laborales y la práctica de la medicina ocupacional.

El ensayo teórico es una metodología de investigación científica que se destaca por la reflexión crítica y la elaboración conceptual sobre un tema específico, sin necesariamente apoyarse en datos empíricos originales. Este tipo de ensayo se basa en el análisis y síntesis de la literatura existente, integrando ideas de diversos autores y teorías para desarrollar una comprensión más profunda del tema en cuestión^18^.

Reconociendo la naturaleza subjetiva de un ensayo teórico^18^^,^^19^, Soares *et al*.^18^ y Meneghetti^19^ destacan que, en los estudios teórico-reflexivos, el pensamiento adquiere autonomía. Esta autonomía se logra al permitir que la subjetividad del ensayista ejerza influencia e importancia en la comprensión del objeto bajo análisis. Así, la apropiación de los conceptos no sigue un enfoque sistemático u organizado, a diferencia del patrón observado en estudios experimentales, que utiliza y articula los conceptos de manera más formal.

En este sentido, optamos por utilizar la plataformización del trabajo y las masculinidades como fundamentos para nuestras reflexiones y soporte de análisis. Las reflexiones resultaron en dos categorías de discusión: “La inserción laboral a través de aplicaciones móviles en Brasil y el modelo de trabajo mediado por algoritmos”; y “ Hombres, salud y motocicletas: la salud ocupacional y el comportamiento masculino laboral en aplicaciones de entrega”.

## RESULTADOS

En la primera sección, delineamos el proceso de plataformización del trabajo en el contexto brasileño, presentando los diversos regímenes laborales existentes en el país y cómo los hombres brasileños se adaptan a este modo de operación. La tesis que orienta nuestro estudio sugiere que los elementos presentes en este modelo de trabajo tienen propensión a la generalización, ejerciendo influencia en las actuales tendencias de explotación capitalista. Vemos la plataformización del trabajo como un enfoque innovador de gestión y control de la fuerza laboral, que se destaca por la consolidación del trabajo bajo demanda, con una dependencia intrínseca de plataformas digitales para la realización de actividades laborales.

En la segunda sección, buscamos en la salud ocupacional una base para los derechos laborales, explorando cómo las masculinidades, moldeadas por comportamientos de riesgo, se entrelazan con la necesidad financiera. En este escenario, la pérdida de derechos laborales, incluida la garantía de condiciones saludables de trabajo, se subyuga a la ilusión de la autonomía laboral y el régimen de jornada de trabajo flexible. Para ello, concebimos la salud ocupacional como un pilar de garantías en el trabajo formal y las masculinidades como elementos que son moldeados y se reflejan en la adhesión a este tipo de servicio por parte del público masculino, lo que se convierte en una posibilidad de intervención para la salud pública.

### La inserción laboral a través de aplicaciones móviles en Brasil y el modelo de trabajo mediado por algoritmos

El cambio histórico en el período postindustrial ha generado la aparición de una nueva estructura social, con un mayor enfoque en el sector de servicios. Esto se debe, principalmente, a la organización del sistema productivo en torno a los principios de maximización de la productividad. Antunes y Praun^13^ destacan que esto inevitablemente hace que el trabajo sea precario debido a la exigencia de un proceso de producción ágil y económico. Además, la precariedad del trabajo coexiste con la falta de derechos laborales que surgen en la era moderna, asociada principalmente a una carga horaria extenuante y a la extrema dificultad de ascenso social y económico debido a la escasez de oportunidades.

En el contexto brasileño, destacamos los avances y retrocesos en el mercado laboral. Durante la llamada “Era Vargas” (1930-1945), se implementaron medidas que permitieron la reglamentación del movimiento obrero y establecieron reglas para el acceso al empleo y a la ciudadanía. Sin embargo, con la llegada de la era neoliberal, que afectó especialmente a los países capitalistas subdesarrollados, hubo una redefinición del polimorfismo del trabajo y una reorganización de la precarización. Uchôa de Oliveira^12^ destaca que la crisis del desarrollismo resultó en la formación de grandes cordones de informalidad y miseria metropolitana, desafiando los avances anteriores en términos de derechos laborales y seguridad social. Como consecuencia, la precarización laboral se convirtió en una realidad preocupante, con muchos trabajadores tuvieron que enfrentar condiciones laborales inestables y salarios inadecuados.

En este contexto, entendemos que las llamadas sociedades de la información presentan una estructura social cada vez más polarizada, en la que los extremos aumentan su participación en detrimento de los sectores medios. A partir de esto, un gran número de trabajadores tiende a crear sus propias oportunidades de ingresos, teniendo que autogestionarse e invertir en sí mismos para volverse empleables. Además, la crisis económica y la necesidad financiera ejercen un peso importante en la toma de decisiones para los trabajadores informales que operan a través de aplicaciones, ya que trabajar como colaboradores de estas empresas se convierte en una solución al desempleo en un mercado altamente excluyente^20^.

Al reflexionar sobre el contexto del empleo vinculado a las plataformas digitales, la formalidad en la contratación de profesionales es inexistente. No se observan posiciones predefinidas ni procesos de selección tradicionales; aparentemente, el simple registro es suficiente para comenzar las actividades laborales. El contrato de trabajo convencional asume, en este entorno, la forma de un contrato de adhesión. Sin embargo, las empresas han tenido éxito al consolidar su presencia en sectores específicos y gestionar grandes contingentes de trabajadores. Además, Abílio *et al.*^3^ destaca que la dinámica de subordinación adopta un enfoque más informal, caracterizado por la falta de definiciones claras o estables con respecto a la jornada laboral, distribución de tareas e incluso la fijación de precios del trabajo.

Vincular estas condiciones a la adhesión a las plataformas de entrega por aplicación, destinadas a los motociclistas muestra, en general, un procedimiento simplificado. Inicialmente, la persona interesada en convertirse en un repartidor realiza su registro en la plataforma, proporcionando información personal, documentos necesarios y detalles sobre su motocicleta. Después de la verificación y aprobación de estos documentos, el motociclista es habilitado en la plataforma. Cuando un usuario solicita una entrega, el algoritmo de la plataforma analiza varios parámetros, como la proximidad del motociclista, el tiempo estimado para la entrega y la demanda en la zona. La entrega se ofrece al motociclista más adecuado, considerando estos criterios. El repartidor tiene la opción de aceptar o rechazar la entrega, lo que le brinda cierta flexibilidad para gestionar su jornada laboral^3^^,^^4^.

Destacamos que la gestión del algoritmo, en teoría, es fundamental para optimizar la eficiencia del servicio. Este manejo tiene en cuenta variables dinámicas, como el tráfico, la demanda en tiempo real y la disponibilidad de los motociclistas. Sin embargo, entendemos que en la era de las tecnologías y la intensificación del ritmo de vida, estas plataformas digitales surgen como un mecanismo seductor que, además de proporcionar rentabilidad para posibles desempleados, pretende ofrecer a los proveedores de servicios un bien precioso: el autocontrol del tiempo.

Adicionalmente, de acuerdo con Abílio^4^, la problemática radica en la ausencia de un mecanismo legal entre los límites y características de este tipo de relación laboral que coloca a los individuos en situaciones de diversas vulnerabilidades sociales. Por lo tanto, la precarización del trabajo en estos escenarios reproduce una cadena de reducción de derechos y garantías de seguro de desempleo que son fuertemente defendidos en el mercado laboral formal. Así, la desarticulación de la clase trabajadora a través del proceso de plataformización del trabajo está en un creciente movimiento de individualización para que el trabajador sea visto solo como un proveedor de servicios y se produzca un aumento de la explotación^3^^,^^4^.

Estos hombres, alejados de las garantías laborales, se encuentran en constante riesgo. Durante la pandemia, las medidas de protección para estas categorías fueron costeadas por cuenta propia y las empresas se limitaron en gran parte a enviar orientaciones para la prevención del contagio. Entre otros riesgos, en un régimen de trabajo conocido como *just in time*^21^, en el cual la gestión se realiza a través de los algoritmos de las aplicaciones, en el que la demanda de trabajo hace que el trabajador esté disponible en cualquier momento para prestar servicios, hay mayores posibilidades de accidentes de tránsito^7^. Según Abílio^3^^,^^4^, esto se debe a que estos trabajadores circulan diariamente por las calles, a menudo a altas velocidades, con el objetivo de reducir el tiempo de espera del destinatario y aumentar su productividad. Por lo tanto, vincularse a las plataformas se convierte en un mecanismo de subsistencia en un escenario económico rezagado, pero al mismo tiempo altamente competitivo. Así, el aumento exponencial de los trabajadores por aplicación refleja la forma de reclasificar la mano de obra, y así la pérdida de derechos se entrelaza con la necesidad financiera^3^^,^^4^.

### Hombres, salud y motocicletas: la salud ocupacional y el comportamiento masculino laboral en aplicaciones de entrega

La década de 1970 marcó el desarrollo social a través de la reforma sanitaria brasileña. Este movimiento propuso nuevas formas de organizar y brindar atención médica a la población que, en tiempos de crisis económica, social y política en el país, diseñó un sistema de salud pública para ampliar la cobertura de la atención médica preventiva y garantizar los derechos sociales^22^. Estos movimientos permitieron una visión ampliada de la salud que también incluyó las condiciones laborales como determinantes del proceso salud-enfermedad.

En cuanto a la salud masculina, el interés de la salud pública por esta temática es relativamente reciente. Según Toneli *et al*.^11^, en Brasil, el primer manuscrito relacionado con la salud masculina se publicó en 1998. En América Latina en su conjunto, estos estudios surgen inspirados por las teorías de género, marcando la emergencia de incorporar a los hombres en los debates sobre la salud como una forma de acercar a estos individuos a los servicios de atención primaria y secundaria^16^^,^^17^. 

De acuerdo con Connel^9^, las masculinidades originadas en la cultura patriarcal y machista potencian prácticas basadas en creencias y valores sobre lo que significa ser masculino. Destacamos que la enfermedad se considera una señal de debilidad que los hombres no reconocen como inherente a su propia condición de vida. Por lo tanto, el hombre se considera intocable, no sujeto a desequilibrios biológicos o enfermedades del cuerpo, exponiéndose así a condiciones de riesgo. Al adoptar el estereotipo masculino, el hombre se siente instintivamente obligado por la sociedad a incorporar ciertos comportamientos, como ser el protector del hogar, responsable del sustento familiar, educación de los hijos y salud de todos. A menudo, el hombre asume estas responsabilidades hacia sus seres queridos y termina olvidándose de sí mismo, descuidando su propia salud^15^^,^^16^^,^^17^.

Para el trabajador brasileño, el trabajo o la falta de él también es un determinante importante de las condiciones de vida y la situación de los trabajadores. Además de generar ingresos, el trabajo posibilita condiciones materiales, inclusión social y la formación de redes de apoyo importantes para el bienestar social. Sin embargo, por ser una condición multifacética, el trabajo como determinante social también puede profundizar las desigualdades y vulnerabilidades que, en consecuencia, agravan el proceso de enfermedad^23^. Así, la salud del trabajador se ha convertido en un campo de prácticas y estrategias interdisciplinarias e interinstitucionales que buscan analizar e intervenir en las condiciones laborales que pueden provocar enfermedades y padecimientos. Esto marca un hito histórico para la salud colectiva en la promoción, prevención y vigilancia de condiciones insalubres y riesgos laborales^24^.

La problemática radica en que, en un escenario de flexibilización laboral y reducción de derechos y garantías constitucionales, esta población se aleja de los principios y objetivos de la salud ocupacional. En 2020, el mundo comenzó a enfrentar la pandemia de covid-19 y, a nivel global, se instó a una amplia gama de trabajadores a realizar teletrabajo. Sin embargo, no todas las actividades podían llevarse a cabo a distancia, como es el caso de los motociclistas que entregan pedidos a través de aplicaciones, una profesión considerada altamente esencial para mantener el distanciamiento social. A pesar de que algunos estudios^12^^,^^25^ defienden la importancia de este tipo de trabajo para reducir los riesgos de transmisión, entendemos que estos trabajadores considerados esenciales ya son social y económicamente vulnerables. Sin embargo, en un estado de calamidad pública, cobraron relevancia debido a la alta demanda de entregas durante la pandemia, sin que hubiera ninguna preocupación por el aislamiento social, ya que se convirtieron en una fuerza para combatir la transmisibilidad del virus mediante su labor considerada esencial.

Durante este período, estos trabajadores estuvieron completamente expuestos al riesgo de contagio por el coronavirus. Atrapados por la necesidad económica, se enfrentaron diariamente a un virus altamente contagioso y a riesgos inherentes a su actividad para establecer una asociación unilateral entre el conductor y la aplicación móvil. Por este motivo, las masculinidades planteadas por Connel^9^ nos llevan a reflexionar sobre cómo el hombre cuida de sus cuerpos, lo que sufre e interfiere directamente en el conjunto de significados adquiridos a lo largo de la vida. Desde las primeras experiencias infantiles que dictan que el hombre debe ser fuerte, sin mostrar sentimientos como dolor y fragilidad, hasta las normas, creencias y valores internalizados, que hacen que el individuo reaccione de manera absolutamente personal a comportamientos de riesgo, influyendo en los cuidados que ofrece a sí mismo, como destacan otros estudios^26^^,^^27^.

El cuidado de sí, en este contexto, se refiere al acto de cuidar de uno mismo, tanto individual como colectivamente, incorporando valores culturales, ambientales, sociales y formativos adquiridos y cultivados a lo largo de la vida. Este cuidado permite que los hombres vivan de manera saludable consigo mismos y con su entorno, quienes también se benefician de esta actitud. Así, el cuidado de sí va más allá de la simple superación de las dicotomías de equilibrio y desequilibrio del cuerpo masculino, ya que se internaliza en las acciones del cuidado saludable, que abarcan todos los aspectos biopsicosociales^26^.

Sin embargo, en el contexto pandémico se puso en evidencia el lado perverso de un régimen de trabajo sin garantías. En un intento de proporcionar subsidios básicos para esta categoría, en el ámbito jurídico, la Justicia Laboral Brasileña determinó el 5 de abril de 2020 que una aplicación de entrega de comida rápida debería garantizar la asistencia financiera a grupos de riesgo o bajo sospecha de contagio, además de proporcionar insumos como alcohol en gel para llevar a cabo sus actividades laborales. Sin embargo, después de la apelación de la empresa, un día después de este dictamen, el Tribunal Regional de Trabajo de la 2ª Región revocó dicha decisión, alegando que no hay relación laboral entre la aplicación y los repartidores^28^.

Por otro lado, los establecimientos comerciales que permanecieran abiertos en el marco de la pandemia estarían obligados a proporcionar máscaras de protección individual de forma gratuita a sus empleados debidamente registrados. La Ley 14019, del 2 de julio de 2020, destaca:

Art. 3º-B. Los establecimientos en funcionamiento durante la pandemia de Covid-19 están obligados a proporcionar de forma gratuita a sus empleados y colaboradores máscaras de protección individual, incluso de fabricación artesanal, sin perjuicio de otros equipos de protección individual establecidos por las normas de seguridad y salud en el trabajo.^29^

En este sentido, comprendemos que la salud del trabajador se vuelve inexistente para la población que opera en el mercado informal, lo cual va en consonancia con lo esperado en el régimen de trabajo flexible del sistema capitalista. La salud ocupacional, que enfatiza la seguridad del entorno laboral y enumera como deber del empleador mantener condiciones de trabajo saludables, sufre en períodos de transferencia de responsabilidades, cuando los empleadores buscan alejarse de los derechos laborales a través de nuevas relaciones laborales.

Desde el punto de vista de la salud pública, cada trabajador debería ser considerado y estar preparado para comprender su protección y entender los riesgos de su actividad laboral. En el contexto de la pandemia, las medidas adoptadas en una acción coordinada por los servicios de medicina ocupacional muestran una reducción significativa de la transmisibilidad en el ámbito laboral, además de una ventaja adicional en la lucha contra el desempleo^30^.

## DISCUSIÓN

La convergencia entre la medicina del trabajo y la creciente plataformización del trabajo emerge como un factor de relevancia incuestionable al analizar los desafíos contemporáneos enfrentados por los trabajadores en entornos laborales caracterizados por la flexibilidad y la informalidad. La dinámica que introduce este modelo de trabajo, definido por el surgimiento de plataformas digitales que conectan proveedores de servicios con demandantes de manera flexible y descentralizada, ha reconfigurado de manera inédita las relaciones laborales. Ante este panorama, la medicina del trabajo desempeña un papel preponderante, ya que los trabajadores involucrados en este paradigma a menudo se enfrentan a condiciones laborales singulares y desafiantes.

La flexibilidad y fragmentación inherentes a la plataformización del trabajo pueden impactar la salud ocupacional de los trabajadores de maneras multifacéticas. La ausencia de un empleo formal a menudo resulta en la falta de beneficios esenciales, como planes de salud, licencias remuneradas y cobertura previsional. En este contexto, respaldado por los ideales de pérdida de garantías de Abílio et al., ^3^, entendemos que la medicina del trabajo, al priorizar la prevención y promoción de la salud, se vuelve imperativa para garantizar que los trabajadores involucrados en este nuevo paradigma tengan acceso a servicios de salud adecuados, monitoreo regular e intervenciones preventivas, siendo un posible medio para fortalecer las políticas laborales para esta población.

Connell^9^ destaca que la población masculina, siguiendo los ideales hegemónicos de fuerza y virilidad, tiende a exponerse a riesgos relacionados con su salud. Además, el trabajo a través de aplicaciones, sin vínculo laboral, a menudo implica jornadas laborales irregulares y prolongadas, ampliando los factores de riesgo como fatiga, estrés y trastornos del sueño. En este contexto, defendemos que la medicina del trabajo, al realizar un análisis profundo y comprensivo de estos patrones, puede concebir estrategias específicas para mitigar estos impactos negativos en la salud de los trabajadores, apoyadas en la cultura masculina tal como describe Connell^9^. 

Reflexionar sobre los hombres que trabajan como repartidores a través de aplicaciones móviles revela el universo del motociclismo y las representaciones de masculinidades. Miranda y Nascimento^8^ describen a los motociclistas con adjetivos como imprudencia, audacia, irresponsabilidad, incivilidad y aprecio por fuertes emociones. Estos factores conforman un perfil que se alinea notablemente con los valores y comportamientos históricamente asociados a lo masculino, según lo propuesto por Connell^9^. En esta reflexión, la imagen del motociclista trasciende las calles, reflejando patrones culturales y sociales que moldean las percepciones sobre la masculinidad.

Los hombres en el ámbito profesional, especialmente aquellos vinculados a ocupaciones consideradas perjudiciales para la salud, encuentran la oportunidad de resaltar su virilidad al destacar la resistencia de sus cuerpos frente a condiciones adversas, enfrentando diariamente la posibilidad de enfermarse y/o poner en riesgo su vida^8^^,^^31^. La concepción de invulnerabilidad, en este contexto, desempeña un papel crucial en la formación de la identidad masculina: la convicción de que “nada les sucede a los hombres” refleja la búsqueda del riesgo como un valor cultural intrínseco, intensificado por los medios de comunicación de masas, especialmente entre los hombres más jóvenes^8^^,^^9^.

En resumen, creemos que la intersección entre la medicina del trabajo y la plataformización del trabajo no solo destaca las complejidades emergentes en las relaciones laborales contemporáneas, sino que también subraya la necesidad urgente de enfoques adaptativos e innovadores por parte de la medicina laboral. El compromiso proactivo de estos profesionales se vuelve esencial para enfrentar los desafíos únicos impuestos por la transformación del panorama laboral, asegurando así la preservación de la salud y el bienestar de los trabajadores en un entorno laboral en constante crecimiento.

Un estudio de revisión^31^ mostró que las principales quejas de los repartidores de aplicaciones al final de su jornada laboral son dolores de espalda y cabeza, problemas circulatorios y fatiga muscular. Además, la exposición a ruidos y la alta probabilidad de accidentes están vinculados a los riesgos inherentes de esta ocupación. En este ensayo teórico-reflexivo, destacamos que, a contramano del sistema capitalista, la salud ocupacional juega un papel decisivo dentro de las empresas para garantizar condiciones más saludables para los trabajadores. Sin embargo, dentro de la informalidad, se encuentra atada de manos. La falacia de tener ingresos en momentos oportunos y ser su propio jefe ha colocado a estos trabajadores en el centro de una crisis sanitaria en la que las ganancias se mantienen por encima de las necesidades de los trabajadores, cualesquiera que estas sean. En ocasiones, la enfermedad puede ser una moneda de cambio barata para la continuidad de este sistema económico y flexible.

En cuanto a las masculinidades en el ámbito de la salud ocupacional y en la dinámica peculiar del trabajo informal, destacamos las construcciones sociales de la masculinidad. Estas a menudo se vinculan a ideales de autonomía, resistencia y autosuficiencia, pudiendo influir en las elecciones profesionales y en la adopción de prácticas laborales. En el contexto de la informalidad, la búsqueda de independencia financiera y autonomía a menudo está moldeada por esta identidad masculina, llevando a los trabajadores a aceptar condiciones laborales precarias en nombre de la preservación de una imagen autónoma y resiliente.

Destacamos la importancia de que la medicina del trabajo dirija su atención a los comportamientos masculinos en el entorno laboral. Reconocer la complejidad de las masculinidades, según propone Connell^9^, se vuelve esencial para entender cómo las construcciones sociales de género influyen en las elecciones y actitudes de los trabajadores, especialmente en la informalidad. La medicina del trabajo, al considerar las sutilezas de los comportamientos masculinos y al crear estrategias más dirigidas al público con un mayor número de trabajadores informales a través de aplicaciones, puede desarrollar enfoques más efectivos y culturalmente sensibles para promover la salud y el bienestar de estos profesionales.

En este escenario, resaltamos la necesidad de reconocer la importancia de la salud de los trabajadores como un pilar fundamental para su protección, independientemente del régimen laboral en el que se encuentren. Por lo tanto, es importante tomar medidas para asegurar que la salud del trabajador sea una prioridad en todas las esferas del mercado laboral, incluido el sector informal. Esta protección se ha centrado en el período de la pandemia, pero debe apuntar a construir una realidad en la que todos los trabajadores puedan realizar sus actividades laborales con dignidad, seguridad y bienestar, en consonancia con los principios fundamentales de los derechos humanos. Solo así podremos avanzar hacia una sociedad más justa e inclusiva, donde la salud y el bienestar de los trabajadores sean verdaderamente valorados.

## CONSIDERACIONES FINALES

La pandemia de la covid-19 afectó la economía internacional, pero tuvo un impacto aún mayor en la población que trabaja en la informalidad. En un intento por encontrar nuevos medios de subsistencia, los hombres sin vínculos laborales encontraron en las aplicaciones para motociclistas de entrega una oportunidad para seguir trabajando. El número de estos trabajadores creció exponencialmente durante la pandemia, y la plataformización del trabajo se consolidó como una oportunidad para convertirse en el propio jefe. El peligro en esta situación radica en encontrar un equilibrio entre los deberes y derechos de estos profesionales, ya que realizan sus funciones en rutinas exhaustivas y dependen de una lógica algorítmica programada para maximizar los beneficios empresariales.

Trabajar como motociclista expone a estos trabajadores a diversas situaciones de riesgo que, en medio de una pandemia, se volvieron servicios esenciales para reducir la transmisibilidad del virus. Sin ningún subsidio financiero para mantenerse aislados, la solución es asumir los propios costos y recorrer la ciudad en busca de un salario para enfrentar la crisis económica que también afecta a esta clase.

Las masculinidades en este contexto también desempeñan un papel importante, ya que los hombres que trabajan como motociclistas enfrentan desafíos específicos en relación con la salud ocupacional. La cultura de la masculinidad a menudo los alienta a asumir riesgos, actuar sin miedo y no mostrar vulnerabilidad. Estos valores pueden influir negativamente en sus decisiones laborales, llevándolos a descuidar su propia salud en aras de la productividad y el cumplimiento de objetivos.

Además, la noción tradicional de “ser proveedor” puede presionarlos a trabajar exhaustivamente y sin pausas, incluso en un contexto pandémico que requiere cuidados adicionales. La búsqueda de independencia financiera, común en algunas representaciones de la masculinidad, puede llevarlos a enfrentar riesgos sin la protección adecuada, como la falta de equipos de seguridad durante las entregas.

Por lo tanto, la salud ocupacional, que da voz y lugar a los trabajadores dentro de un sistema capitalista, no se ajusta a este modo de trabajo. Sin garantías y en jornadas extenuantes, estos individuos no tienen ningún apoyo por parte de la medicina preventiva ni beneficios salariales adicionales por el constante riesgo en el que viven. En este escenario, la salud es un determinante en varios aspectos de la vida humana y también puede ser influenciada por el trabajo. En contraste con el movimiento de reforma sanitaria brasileña, que fortaleció las políticas de salud pública y sociales y buscó, por ley, garantizar medios de subsistencia mínimos en el mercado laboral, la flexibilización de los regímenes de trabajo viene a romper esas garantías y fortalecer el proceso de reducción de derechos.

Es importante señalar que estas elecciones teóricas y metodológicas imponen limitaciones analíticas al estudio, un aspecto que debe considerarse en la interpretación de las reflexiones presentadas en este manuscrito.
